# Supplementary material for: Translation velocity determines the efficacy of engineered suppressor tRNAs on pathogenic nonsense mutations
Source: Nat Commun. 2024 Apr 5;15:2957. doi: 10.1038/s41467-024-47258-9 (PMC10997658; doi:10.1038/s41467-024-47258-9)
Supplement: Supplementary file 1 — Supplementary Information [file 41467_2024_47258_MOESM1_ESM.pdf]

Supplementary Information

Translation velocity determines the efficacy of engineered suppressor tRNAs on pathogenic nonsense mutations

Nikhil Bharti[1,#], Leonardo Santos[1,#], Marcos Davyt[1], Stine Behrmann[1], Marie Eichholtz[1], Alejandro Jimenez-Sanchez[1], Jeong S. Hong[2,3], Andras Rab[2,3], Eric J. Sorscher[2,3], Suki Albers[1]*, Zoya Ignatova[1]*

[1]Institute of Biochemistry and Molecular Biology, University of Hamburg, 20146 Hamburg. Germany.

[2]Department of Pediatrics, School of Medicine, Emory University, Atlanta, GA 30322, USA.

[3]Children's Healthcare of Atlanta, Atlanta, GA 30322, USA

[#]equally contributed

*Correspondence to: Zoya Ignatova, email: zoya.ignatova@uni-hamburg.de and Suki Albers: suki.albers@uni-hamburg.de

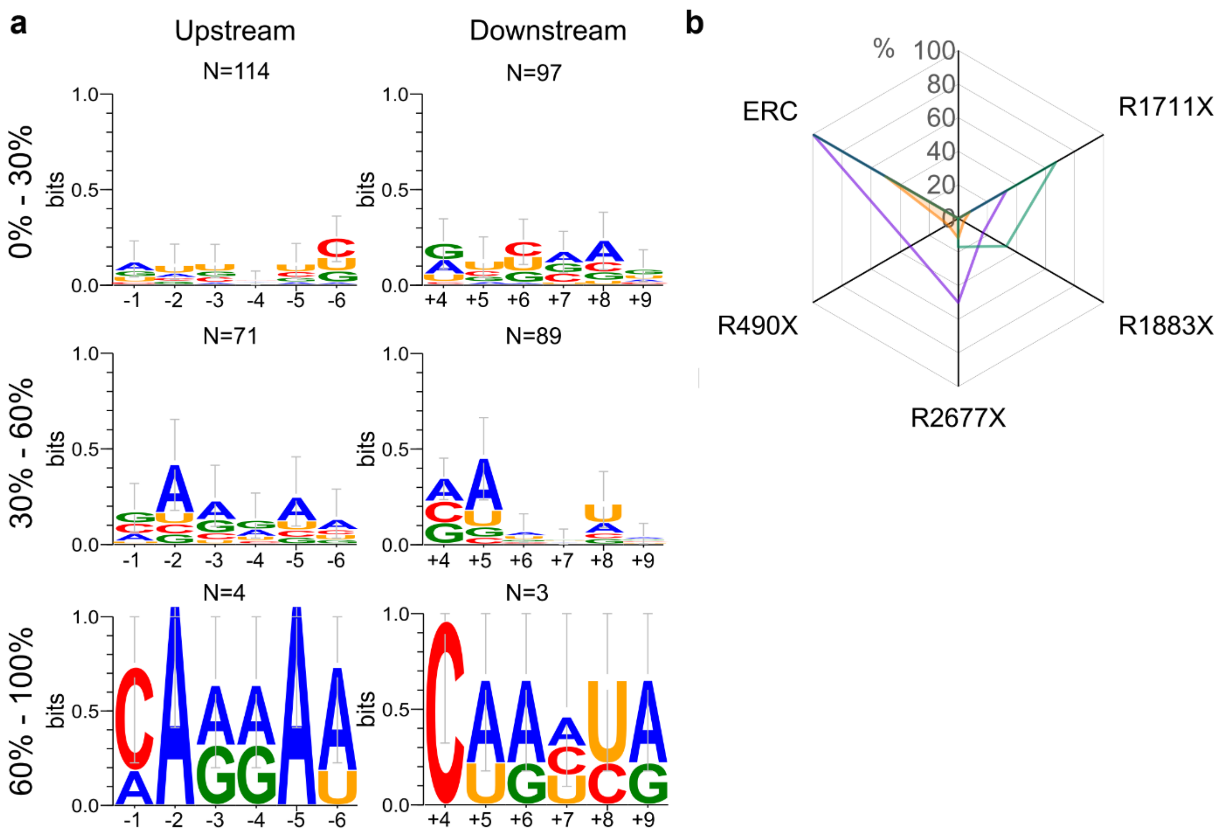

Supplementary Fig. 1. The sup-tRNA efficiency at PTCs associated with PCD does not correlate with the sequence similarity to the ERC. a, Sequence logos of the 6 nt-sequence upstream (left column) or downstream (right column) of pathogenic PTCs in all PCD-associated genes (Supplementary Table 1). Sequences are grouped in three groups based on their resemblance to the efficient readthrough context (ERC) (percentage on left margin). N, number of PTCs in each group. **b**, Spider diagram for specific PTCs (X=UGA) in *RSPH4A* and *DNAH5* (orange filled) displaying the experimentally measured readthrough efficiency (Fig. 1b) and their resemblance to the upstream (green line) and downstream (purple line) sequence. The readthrough efficiency of the ERC sequence is 39%. Source data are provided as a Source Data file.

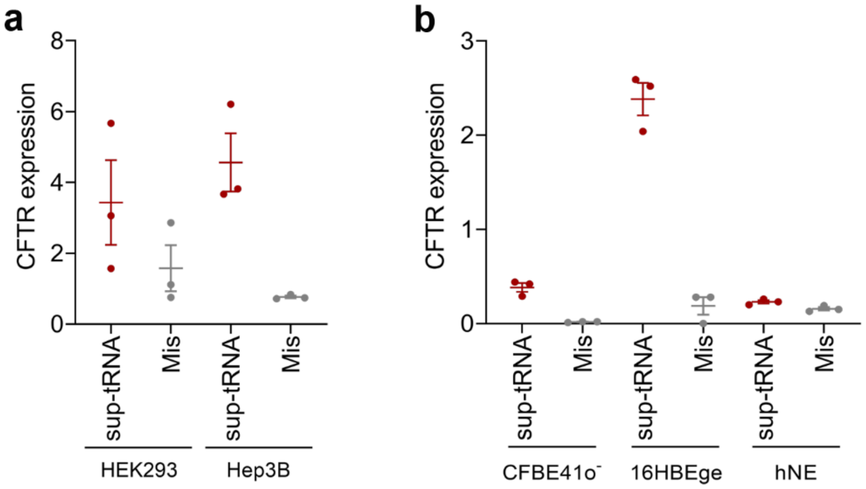

Supplementary Fig. 2. Cell-dependent R1162X PTC (X=UGA) suppression in *CFTR*. a, Sup-tRNA^{Ser} (red) efficacy for restoring expression of full-length R1162X-CFTR expressed on a plasmid cDNA in HEK293 and Hep3B cells. **b**, Sup-tRNA^{Arg} (red) efficacy for restoring expression of full-length R1162X-CFTR expressed from plasmid cDNA in CFBE41o− cells or endogenously expressed (i.e. with all introns and exons) in human bronchial epithelial cells (16HBEge R1162X/-) or human nasal epithelia (hNE, R1162X/R1162X) derived from a CF patient homozygous for R1162X mutation. The sup-tRNA^{Arg} is the variant tRT5 which introduces Arg at the affected codon[2]. Mis, mismatch tRNA (gray in **a**, **b**) that does not pair to the PTC. Suppression activity was monitored by automated immunoblotting (JESS system) using monoclonal CFTR-NBD2 antibody. Data in **b** are from[2]. Data in **a** and **b** are means ± s.e.m. (n=3 independent replicates). Source data are provided as a Source Data file.

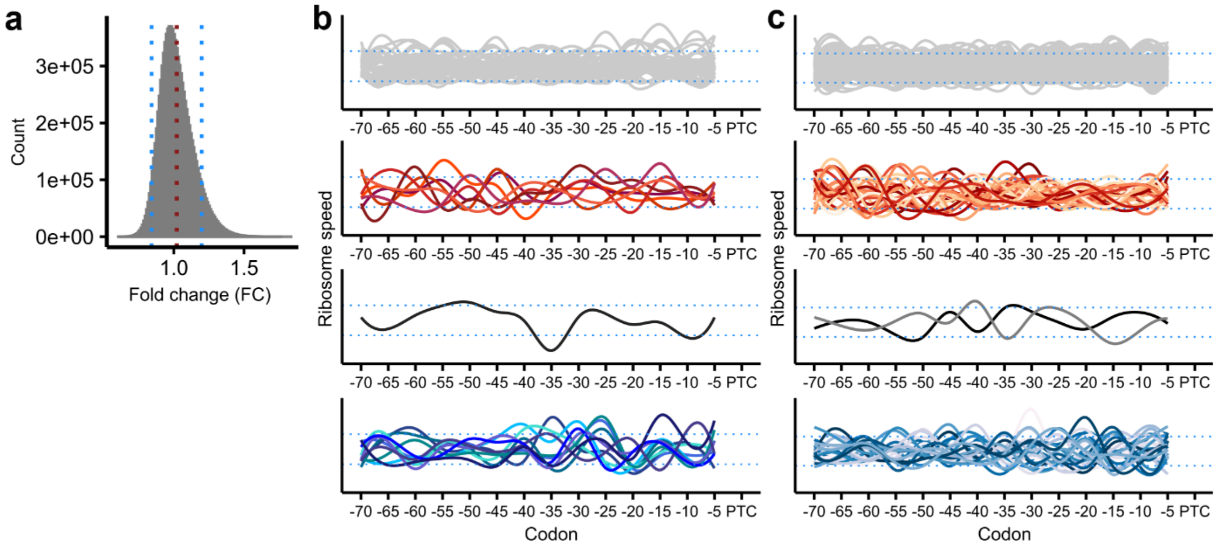

Supplementary Fig. 3. Sequences upstream of PTCs show different translation profiles. a, The average velocity across the entire transcriptome follows normal distribution. The average translation speed of all possible consecutive 5-codon segments was related to the average translation speed of a given transcript and represented as fold-change. Blue dotted lines designate 1.5σ. Red dotted line designates the mean of the average velocity. **b, c**, Translation velocity profiles of the region upstream of PTCs in *CFTR* (**b**) and genes associated with PCD (**c**) (Supplementary Table 1). Blue horizontal dotted lines designate the ±1.5σ (panel **a**). PTCs with a smooth translation profile (i.e. within 1.5σ (gray shades); PTCs with inversion of the translation speed (i.e. > 1.5σ) and related to the distance to PTC as close (blue shades, 5-35 codons), middle (dark gray shades, 20-45 codons) and distant (red shades, 35-70 codons). Source data are provided as a Source Data file.

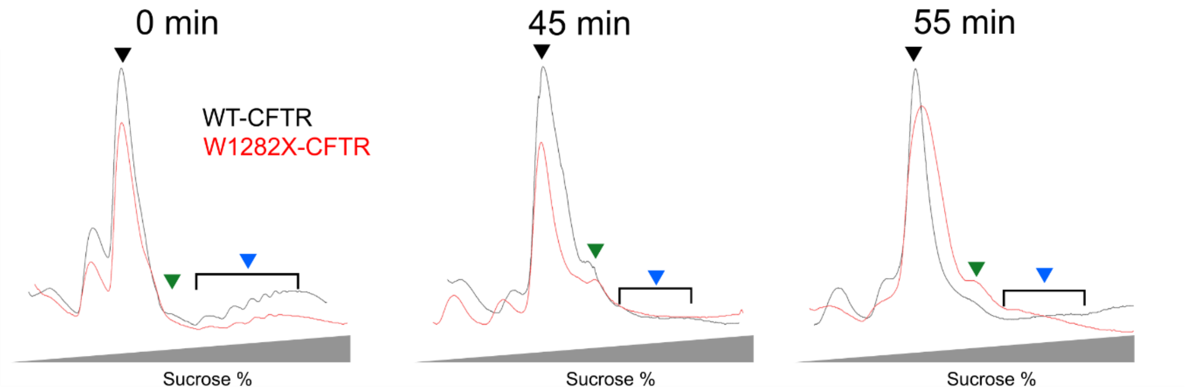

Supplementary Fig. 4. Establishing the conditions for obtaining nuclease-resistant di-ribosome (disome) fraction. Polysomes of Calu3 cells expressing WT-CFTR (black) and W1282X-CFTR (red) were collected at time zero and treated for different times with RNase I. Monosomes (black triangle), disomes (green triangle) and polysomes (blue triangle) were separated on sucrose gradients (horizontal gray triangle designates the sucrose gradient). Disomes that are separated from one another were detectable at shorter RNase I treatments, but disappear at prolonged incubation. Stable nuclease-resistant disomes in W1282X-*CFTR* expressing cells were seen at 55 min. The experiment served as a quality check for the presence of nuclease-resistant disomes and hence, was performed as a single experiment. Source data are provided as a Source Data file.

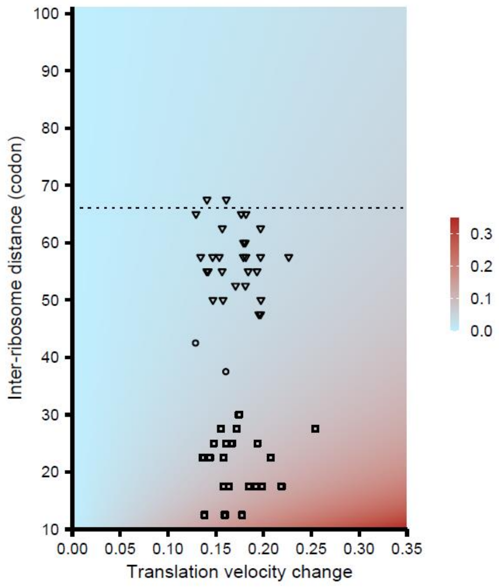

Supplementary Fig. 5. Predicting the collision probability for pathologic nonsense mutations associated with PCD pathology. PTCs in genes associated with PCD pathology were categorized based on velocity changes (or inversions) related to their distance from PTCs (single translation velocity profiles in Supplementary Fig. 3c): close (5-35 codons, squares), middle (20-45 codons, circle) and distant (35-70 codons, triangles). For details of the collision probability model at PTCs see Fig. 6 The horizontal dashed line denotes the transcriptome-wide average inter-ribosomal distance (IRD) of 66 codons. The identity of the mutations is provided as a Source Data file.

Supplementary Table 1. Frequency of the PTC mutations in two pathologies CF and PCD. CF is a monogenic disease with mutations in *CFTR* are associated with CF, while PCD is linked to mutations in several genes. Mutations are color coded based on the translational profiles (Supplementary Fig. 3): gray, mutations with a smooth translation profile (i.e. within 1.5σ); PTCs with inversion of the translation speed (i.e. > 1.5σ) and related to the distance to PTC as close (blue, 5-35 codons), middle (black, 20-45 codons) and distant (red, 35-70 codons).

Disease	Gene	PTC mutations
PCD	*CCDC39*	CCDC39_E222X, Q165X, R96X, E1117X, E891X, K1033X, Q139X, Q142X, Q439X, Q619X, Q651X, Q883X, R449X, Y1118X<br>C134X, Y742X
	DNAH11	C182X, E1070X, E1074X, E1790X, E2089X, K3152X, K4078X, K496X, L3794X, Q1075X, Q1523X, Q1740X, Q2427X, Q2916X, Q3442X, Q3597X, Q3730X, Q4233X, Q4391X, R1045X, R1182X, R1445X, R1480X, R1541X, R2044X, R2082X, R2243X, R2845X, R2900X, R3160X, R3236X, R3346X, R3350X, R3809X, R3841X, R3845X, R4104X, R4357X, R4359X, R857X, S2169X, W1041X, W1733X, W4336X, W453X, Y2870X, Y3420X, Y4121X<br>E3175X, R1865X
	DNAH5	C1679X, C2750X, C3160X, E117X, E1315X, E3293X, E4080X, E912X, E918X, E91X, G3644X, K3094X, L887X, Q1087X, Q1162X, Q1177X, Q1317X, Q1450X, Q1687X, Q1828X, Q2426X, Q2723X, Q2802X, Q3232X, Q3260X, Q3267X, Q3462X, Q3609X, Q3846X, Q4303X, Q4325X, Q4387X, Q610X, Q639X, Q684X, Q845X, R1454X, R2013X, R2228X, R224X, R2677X, R2795X, R3000X, R3042X, R3116X, R3168X, R333X, R3481X, R3909X, R4476X, R4496X, R478X, R742X, S1721X, S1733X, S2989X, S3022X, W2822X, W3969X, W4471X, W4481X, Y1304X,Y2607X, Y3133X ,Y537X<br>R2639X, R2772X, R618X, W2539X, W327X, W4036X, W4096X, W4206X, Y1706X, Y1886X, Y1923X, Y84X<br>S2103X<br>C256X, E1756X, E3981X, K1853X, K3676X, Q1401X, Q4089X, R1711X, R1761X, R3620X, R4429X, S3935X, W2345X, W4271X, Y3178X, Y4165X, Y442X
	CCDC40	DNAH11_R2002X, R4395X, S262X, Y1039X<br>E187X, E329X, E352X, E4211X, L3122X, Q1362X, Q4196X, R1995X, R2255X<br>R321X<br>E426X
	RSPH4A	R490X
CF	*CFTR*	R792X, E831X, R785X, R764X, E822X, S466X, S1255X, L1254X, Y913X, R709X, R553X, E379X, G550X, E664X, Q1412X, Q1411X, R75X, S912X, Y275X, C276X, W846X, R851X, S489X, K710X, W401X, G673X, W1204X, Y849X, W496X, E1371X, S1196X, R1102X, C524X, W1098X, W216X, E585X, E193X, Q525X, Q715X, Q720X, Q220X, Q414X, Q1042X, Q98X, L88X, G330X, X1481X<br>R1162X, Q1382X, Q685X, W1089X, Q890X, Y1092X, W882X<br>R1158X<br>W1282X, G745X, Q1330X, Q493X, W1145X<br>S434X, E1104X, G542X, E656X, E92X

Supplementary Table 2: Summary of the nucleotide and amino acid sequence context of the PTCs experimentally analyzed in this study. These nucleotide sequences were fused downstream of the start codon of FLuc yielding the PTC-FLuc variants (Fig. 1). The PTC is underlined and is flanked by seven codons each site.

Disease	Gene	Mutation	PTC	Nucleotide and amino acid sequence of PTC context
PCD	*DNAH5*	R1711X	UGA	ACTGGGTACTTGGAGAAAAAATGACTGTGCTTTCCTCGGTTTTTC
				-T--G--Y--L--E--K--K--X--L--C--F--P--R--F--F-
PCD	*DNAH5*	R1883X	UGA	AGGGATCTGAGTTCCACGGAATGAGTGAAATACGAGACTCTGATT
				-R--D--L--S--S--T--E--X--V--K--Y--E--T--L--I-
PCD	*DNAH5*	R2677X	UGA	CAGGTTACGAATGAGATAGTGTGACAGCTGATGGAACAAAATGGA
				-Q--V--T--N--E--I--V--X--Q--L--M--E--Q--N--G-
PCD	*RSPH4A*	R490X	UGA	TATTTACGAGCACAAATTGCCTGAATTTCAGCAGGAACCCACGTC
				-Y--L--R--A--Q--I--A--X--I--S--A--G--T--H--V-
CF	*CFTR*	S466X	UGA	TCCACTGGAGCAGGCAAGACTTGACTTCTAATGATGATTATGGGA
			UAG	TCCACTGGAGCAGGCAAGACTTAGCTTCTAATGATGATTATGGGA
			UAA	TCCACTGGAGCAGGCAAGACTTAACTTCTAATGATGATTATGGGA
				-S--T--G--A--G--K--T--X--L--L--M--M--I--M--G-
		W846X	UGA	AGCATACCAGCAGTGACTACATGAAACACATACCTTCGATATATT
			UAG	AGCATACCAGCAGTGACTACATAGAACACATACCTTCGATATATT
			UAA	AGCATACCAGCAGTGACTACATAAAACACATACCTTCGATATATT
				-S--I--P--A--V--T--T--X--N--T--Y--L--R--Y—I-
		W1204X	UGA	CACGTGAAGAAAGATGACATGTGACCCTCAGGGGGCCAAATGACT
			UAG	CACGTGAAGAAAGATGACATGTAGCCCTCAGGGGGCCAAATGACT
			UAA	CACGTGAAGAAAGATGACATGTAACCCTCAGGGGGCCAAATGACT
				-H--V--K--K--D--D--M--X--P--S--G--G--Q--M—T-
		C524X	UGA	TACAGAAGCGTCATCAAAGCATGACAACTAGAAGAGGACATCTCC
				-Y--R--S--V--I--K--A--X--Q--L--E--E--D--I--S-
		G542X	UGA	GAGAAAGACAATATAGTTCTTTGAGAAGGTGGAATCACACTGAGTC
				-E--K--D--N--I--V--L--X--E--G--G--I--T--L--S-
		R553X	UGA	ATCACACTGAGTGGAGGTCAATGAGCAAGAATTTCTTTAGCAAGA
				-I--T--L--S--G--G--Q--X--A--R--I--S--L--A—R-
		R1162X	UGA	AGCTTGATGCGATCTGTCAGCTGAGTCTTTAAGTTCAATGACATGC
				-S--L--M--R--S--V--S--X--V--F--K--F--N--D--M-
		W1282X	UGA	GATTCAATAACTTTGCAACAGTGAAGGAAGCCTTTGGAGTGATACC
				-D--S--I--T--L--Q--Q--X--R--K--P--L--E--S--Y-
		W57X	UAG	TCTGAAAAATTGGAAAGAGAATAGGATAGAGAGCTGGCTTCAAAGG
				-S--E--K--L--E--R--E--X--D--R--E--L--A--S--K-
		E60X	UAG	TTGGAAAGAGAATGGGATAGATAGCTGGCTTCAAAGAAAAATCCT
				-L—E--R--E--W--D--R--X--L--A--S--K--K--N--P-
		Y275X	UAG	AATATCCAATCTGTTAAGGCATAGTGCTGGGAAGAAGCAATGGAA
				-N--I--Q--S--V--K--A--X--C--W--E--E--A--M--E-
		Q493X	UAG	GGAAGAATTTCATTCTGTTCTTAGTTTTCCTGGATTATGCCTGGC
				-G--R--I--S--F--C--S--X--F--S--W--I--M--P--G-
		E813X	UAG	ATTAACGAAGAAGACTTAAAGTAGTGCTTTTTTGATGATATGGAG
				-I--N--E--E--D--L--K--X--C--F--F--D--D--M--E-
		Y122X	UAA	GAGGAACGCTCTATCGCGATTTAACTAGGCATAGGCTTATGCCTT
				-E--E--R--S--I--A--I--X--L--G--I--G--L--C--L-
		S489X	UAA	ATTAAGCACAGTGGAAGAATTTAATTCTGTTCTCAGTTTTCCTGG
				-I--K--H--S--G--R--I--X--F--C--S--Q--F--S--W-
		E585X	UAA	CTAGATGTTTTAACAGAAAAATAAATATTTGAAAGCTGTGTCTGT
				-L--D--V--L--T--E--K--X--I--F--E--S--C--V--C-
		Q685X	UAA	TCCTGGACAGAAACAAAAAAATAATCTTTTAAACAGACTGGAGAG
				-S--W--T--E--T--K--K--X--S--F--K--Q--T--G--E-
		Q715X	UAA	ATACGAAAATTTTCCATTGTGTAAAAGACTCCCTTACAAATGAAT
				-I--R--K--F--S--I--V--X--K--T--P--L--Q--M--N-
		Q1411X	UAA	ATAGAAGCAATGCTGGAATGGTAACAATTTTTGGTCATAGAAGAG
				-I--E--A--M--L--E--W--X--Q--F--L--V--I--E--E-

Supplementary Table 3: Nucleotide and amino acid sequence of the wild-type eW1282X and smW1282X. 35 codons upstream and 3 codons downstream of the respective PTC were fused downstream of the start codon of FLuc yielding PTC-FLuc variants (Fig. 4b). The PTC is underlined. Changed nucleotides are highlighted blue

Mutation	Nucleotide sequence of the PTC context
eW1282X	GGATCAGGGAAGAGTACTTTGTTATCAGCTTTTTTGAGACTACTGAACACTGAAGGAGAAATCCAGATCG ATGGTGTGTCTTGGGATTCAATAACTTTGCAACAGTGAAGGAAGCCT
smW1282X	GGTTCGGGCAAGAGTACTCTACTATCAGCTTTCTTGAGACTACTGAACACTGAAGGTGAAATCCAGATCG ATGGTGTGTCTTGGGATTCAATAACTTTGCAACAGTGAAGGAAGCCT
