# Supplementary material for: Translation velocity determines the efficacy of engineered suppressor tRNAs on pathogenic nonsense mutations
Source: Nat Commun. 2024 Apr 5;15:2957. doi: 10.1038/s41467-024-47258-9 (PMC10997658; doi:10.1038/s41467-024-47258-9)
Supplement: Supplementary file 3 — Reporting Summary [file 41467_2024_47258_MOESM3_ESM.pdf]

nature portfolio

Corresponding author(s): Zoya Ignatova

Last updated by author(s): Mar 6, 2024



# Reporting Summary

Nature Portfolio wishes to improve the reproducibility of the work that we publish. This form provides structure for consistency and transparency in reporting. For further information on Nature Portfolio policies, see our Editorial Policies and the Editorial Policy Checklist.

## Statistics

For all statistical analyses, confirm that the following items are present in the figure legend, table legend, main text, or Methods section.

| n/a | Confirmed | |
|---|---|---|
| ☐ | ☒ | The exact sample size (*n*) for each experimental group/condition, given as a discrete number and unit of measurement |
| ☐ | ☒ | A statement on whether measurements were taken from distinct samples or whether the same sample was measured repeatedly |
| ☐ | ☒ | The statistical test(s) used AND whether they are one- or two-sided<br>*Only common tests should be described solely by name; describe more complex techniques in the Methods section.* |
| ☒ | ☐ | A description of all covariates tested |
| ☐ | ☒ | A description of any assumptions or corrections, such as tests of normality and adjustment for multiple comparisons |
| ☐ | ☒ | A full description of the statistical parameters including central tendency (e.g. means) or other basic estimates (e.g. regression coefficient) AND variation (e.g. standard deviation) or associated estimates of uncertainty (e.g. confidence intervals) |
| ☐ | ☒ | For null hypothesis testing, the test statistic (e.g. *F*, *t*, *r*) with confidence intervals, effect sizes, degrees of freedom and *P* value noted<br>*Give P values as exact values whenever suitable.* |
| ☒ | ☐ | For Bayesian analysis, information on the choice of priors and Markov chain Monte Carlo settings |
| ☒ | ☐ | For hierarchical and complex designs, identification of the appropriate level for tests and full reporting of outcomes |
| ☒ | ☐ | Estimates of effect sizes (e.g. Cohen's *d*, Pearson's *r*), indicating how they were calculated |

*Our web collection on statistics for biologists contains articles on many of the points above.*

## Software and code

Policy information about availability of computer code

| | |
|---|---|
| Data collection | Bulk activity (luciferase) measurements: Spark microplate reader, SparkControl Version 2.3 (Tecan).<br>Immunoblots: Jess capillary electrophoresis system (JESS, ProteinSimple) |
| Data analysis | Immunoblot quantification: Compass for SW Version 6.0.0 (Jess, ProteinSimple) - software provided with the JESS instrument |

For manuscripts utilizing custom algorithms or software that are central to the research but not yet described in published literature, software must be made available to editors and reviewers. We strongly encourage code deposition in a community repository (e.g. GitHub). See the Nature Portfolio guidelines for submitting code & software for further information.

## Data

Policy information about availability of data

All manuscripts must include a data availability statement. This statement should provide the following information, where applicable:

- Accession codes, unique identifiers, or web links for publicly available datasets
- A description of any restrictions on data availability
- For clinical datasets or third party data, please ensure that the statement adheres to our policy

All data are available in the main text or the supplementary materials.
Ribosome profiling data (Ribo-seq) from human CFBE41o- cells are available at the Gene Omnibus (GEO) under the accession numbers GSE74365.

## Research involving human participants, their data, or biological material

Policy information about studies with human participants or human data. See also policy information about sex, gender (identity/presentation), and sexual orientation and race, ethnicity and racism.

| | |
|---|---|
| Reporting on sex and gender | N.A. |
| Reporting on race, ethnicity, or other socially relevant groupings | N.A. |
| Population characteristics | N.A. |
| Recruitment | N.A. |
| Ethics oversight | N.A |

Note that full information on the approval of the study protocol must also be provided in the manuscript.

# Field-specific reporting

Please select the one below that is the best fit for your research. If you are not sure, read the appropriate sections before making your selection.

☒ Life sciences ☐ Behavioural & social sciences ☐ Ecological, evolutionary & environmental sciences

For a reference copy of the document with all sections, see nature.com/documents/nr-reporting-summary-flat.pdf

# Life sciences study design

All studies must disclose on these points even when the disclosure is negative.

| | |
|---|---|
| Sample size | No statistical methods were used to predetermine sample size. Sample size is stated in figure legends. Experiments were designed to detect differences greater than 10% at a significance of p<0.05. |
| Data exclusions | No data were excluded. |
| Replication | Experiments were reproduced in multiple independent biological replicates (at least three times) with similar and consistent results. |
| Randomization | All samples were independently measured in a controlled manner, namely the control (treated with mismatch tRNA) and the experimental conditions (treatment with sup-tRNA) were performed in parallel. |
| Blinding | Blinding is not relevant for the study; the experiments were performed by different co-authors. |

# Reporting for specific materials, systems and methods

We require information from authors about some types of materials, experimental systems and methods used in many studies. Here, indicate whether each material, system or method listed is relevant to your study. If you are not sure if a list item applies to your research, read the appropriate section before selecting a response.

### Materials & experimental systems

| n/a | Involved in the study | |
|---|---|---|
| ☐ | ☒ | Antibodies |
| ☐ | ☒ | Eukaryotic cell lines |
| ☒ | ☐ | Palaeontology and archaeology |
| ☒ | ☐ | Animals and other organisms |
| ☒ | ☐ | Clinical data |
| ☒ | ☐ | Dual use research of concern |
| ☒ | ☐ | Plants |

### Methods

| n/a | Involved in the study | |
|---|---|---|
| ☒ | ☐ | ChIP-seq |
| ☒ | ☐ | Flow cytometry |
| ☒ | ☐ | MRI-based neuroimaging |

## Antibodies

| | |
|---|---|
| Antibodies used | 1) anti CFTR-NBD2 antibody (#596, Lot #596TJ03182012, 1:100 dilution, John R. Riordan and Tim Jensen, University of North Carolina, Chapel Hill, USA) |

| | |
|---|---|
| | 2) rabbit polyclona anti- ZNF598 antibody (1:50 dilution, abcam ab80456. Lot:GR32971 30-2)<br>3) rabbit anti-GCN1L1 antibody (1:50 dilution, Bethyl Laboratories, cat# A301-843A) |
| Validation | For Jess-Immunoblot experiments, we tested different dilutions in the range 1:10-1:250 which is recommended by the manufacturer. The optimal dilution was 1:50 (for anti-ZNF598 and antiGCN1L1) and 1:100 (for anto CFTR-NBD2). |

## Eukaryotic cell lines

Policy information about cell lines and Sex and Gender in Research

| | |
|---|---|
| Cell line source(s) | Human Hep3B (HB-8064) and HEK293 were obained from ATCC/DSM.<br>CFBE41o- laboratory collection; originally received from Dr. Karl Kunzelmann, University of Regensburg, Germany) and Dr. Dieter Gruenert (University of California, San Francisco, CA).<br>16HBEge cells ( R1162X/-) and primary hNE(R1162X/R1162X): received from the Cystic Fibrosis Foundation Therapeutics Lab.<br>Calu3 expressing three copies of WT-CFTR and W1282X-CFTR was created by Jeong S. Hong and Andras Rab, Emory Univ, Atlanta, USA. |
| Authentication | CFBE41o- was tested for the deletion of WT-CFTR (by sequencing and immunoblot).<br>Successful gene editing of Calu3 was validated with next-generation sequencing, mutation specific quantitative PCR and for the W1282X-CFTR gene by W1282X-directed ddPCR. For Hep3B and HEK293 authentification was provided by ATCC; for 16HBEge cells ( R1162X/-) and the primary hNE(R1162X/R1162X) autontificaiton was provided by the Cystic Fibrosis Foundation Therapeutics Lab |
| Mycoplasma contamination | Cells were tested at arrival for mycoplasma contamination using Venor GeM PCR-based detection kit (Merck) and the tests were negative. Tested aliquots at low passage number were stored in liquid nitrogen. Cell lines in culture are regularly (every 6 mo. or by indication) tested for mycoplasma contamination and they all tested negative. |
| Commonly misidentified lines<br>(See ICLAC register) | No commonly misidentified cell lines were used. |
